# Single-Step Chromosomal Engineering Integrates Biocontainment and Therapeutic Function for Regulatory-Oriented Live Biotherapeutic Chassis Design

**DOI:** 10.3390/pharmaceutics18080915

**Published:** 2026-07-24

**Authors:** Gabriela Christina Kuhl, Ciarán Devoy, Munawar Abbas, Emilene Da Silva Morais, Mark Tangney

**Affiliations:** 1 Cancer Research @UCC, College of Medicine and Health, University College Cork, 4th Floor, Western Gateway Building, Western Road, T12 K8AF Cork, Ireland; 2 APC Microbiome Ireland, University College Cork, T12 YT20 Cork, Ireland

**Keywords:** live biotherapeutic products, auxotrophy, genetic stability, bacterial DNA delivery, bactofection, genome engineering, synthetic biology, listeriolysin O, microbial chassis, biosafety

## Abstract

**Background:** Live biotherapeutic products (LBPs) require robust genetic stability and effective biocontainment to support safe clinical translation and regulatory acceptance. **Aim:** This study presents a single-step chromosomal engineering strategy that integrates auxotrophy-mediated biocontainment with therapeutic gene insertion to support regulatory-oriented live biotherapeutic chassis design. **Methods:** A no-SCAR genome-editing approach combining CRISPR/Cas9 and λ-Red recombineering was used to generate an *Escherichia coli* MG1655 Δ*ilvC*::*hlyA* strain by replacing *ilvC* with the *hlyA* gene encoding listeriolysin O. Chromosomal and episomal expression systems were compared for auxotrophy, growth, haemolytic activity, plasmid stability, and intracellular DNA delivery to RAW 264.7 macrophages. **Results:** Auxotrophy was successfully established and restored by branched-chain amino acid supplementation. Chromosomal integration preserved haemolytic activity and bacterial growth while improving long-term genetic stability and plasmid maintenance compared with episomal expression. Both systems supported intracellular DNA delivery, whereas the chromosomal construct showed improved host-cell preservation under higher bacterial challenge. **Conclusions:** This proof-of-concept study supports the feasibility of using a single-step chromosomal engineering strategy to combine intrinsic biocontainment with therapeutic-gene integration in an engineered bacterial chassis.

## 1. Introduction

The use of genetically engineered bacteria as live biotherapeutic products (LBPs) has emerged as a promising platform for delivering therapeutic payloads, modulating immune responses, and influencing host–microbe interactions [1,2,3,4]. LBPs are gaining traction in clinical development due to their ability to colonise specific niches and provide sustained delivery of biologics [5,6,7,8,9]. However, their application is tightly regulated because of biosafety concerns, including uncontrolled proliferation, horizontal gene transfer, and unintended environmental persistence [10,11].

Despite substantial advances in synthetic biology and microbial engineering, LBP development remains constrained by persistent challenges in genetic stability, manufacturing consistency, and predictable in vivo performance, which collectively limit reliable clinical translation [2,12]. Current LBP platforms frequently rely on episomal plasmids that exhibit heterogeneous copy number, poor long-term stability, and rapid loss in the absence of antibiotic selection [13,14]. Continued dependence on antibiotic resistance markers for plasmid maintenance is increasingly incompatible with regulatory expectations for genetically modified microorganisms intended for human administration, as reflected in EU Directive 2009/41/EC and emerging FDA guidance for live microbial therapeutics [10,15]. Furthermore, many engineered strains lack intrinsic containment mechanisms, raising concerns regarding environmental persistence and horizontal gene transfer, both of which represent critical biosafety considerations during regulatory evaluation [3,16]. Consequently, existing LBP engineering approaches often address therapeutic gene expression, genetic stability, and biosafety as independent engineering objectives, frequently requiring multi-step engineering workflows that introduce additional genetic elements, increase metabolic burden, and complicate compliance with quality-by-design principles [6,13]. Therefore, there remains a need for streamlined bacterial chassis that integrate therapeutic function, genetic stability, and intrinsic biocontainment within a unified engineering strategy, providing a regulatory-oriented framework for the development of next-generation LBPs [2,12].

Although substantial progress has been made in engineering bacterial delivery systems, most current strategies address therapeutic gene expression, genetic stability, and biosafety as independent engineering objectives [12,13,15]. Consequently, bacterial platforms that simultaneously integrate stable chromosomal therapeutic gene expression, intrinsic biocontainment, and sustained functional performance within a single engineering strategy remain limited [13,15]. Addressing these complementary design requirements through rational genome engineering may facilitate the development of genetically stable and biosafe bacterial chassis while simplifying manufacturing and supporting translational development under evolving regulatory expectations.

More broadly, recent advances in therapeutic development have highlighted the importance of integrating mechanistic and systems-level approaches to improve the understanding, safety, and translational potential of emerging therapeutic strategies. For example, integrative microbial and hepatic biotransformation coupled with network pharmacology has been used to elucidate the therapeutic mechanisms of complex herbal products, while metabolomics has provided comprehensive insights into the molecular mechanisms underlying neuroregeneration after traumatic brain injury [13]. Although these studies were performed in different therapeutic contexts, they illustrate the growing importance of systems-level methodologies for improving mechanistic understanding and supporting translational development. This broader perspective is consistent with the engineering philosophy adopted in the present study, in which biosafety, genetic stability, and therapeutic functionality are considered together during bacterial chassis development [17,18].

A widely adopted strategy to mitigate these biosafety risks is the engineering of microbial auxotrophy, whereby bacterial cells depend on externally supplied metabolites for growth [19]. Disruption of genes involved in essential biosynthetic pathways generates strains that survive only under supplemented conditions, thereby reducing ecological fitness and limiting persistence outside controlled environments [3]. As an intrinsic biocontainment strategy, auxotrophy is well aligned with the environmental protection principles established under EU contained-use legislation [20], making it an attractive approach for the development of regulatory-oriented live biotherapeutic chassis.

Within the European Union (EU), genetically modified LBPs are regulated under an integrated framework that includes Directive 2009/41/EC on the contained use of genetically modified microorganisms, Directive 2001/18/EC on deliberate environmental release, and Regulation (EC) No 1394/2007 governing Advanced Therapy Medicinal Products (ATMPs), including Gene Therapy Medicinal Products (GTMPs) [20,21,22]. In parallel, agencies such as the FDA, EMA, and ICH increasingly classify LBPs as biological medicinal products that require rigorous demonstration of genetic stability, biosafety, environmental containment, and quality control throughout development and manufacturing [1,14,23]. These regulatory expectations highlight the need for microbial chassis that incorporate intrinsic biocontainment, stable therapeutic gene expression, and antibiotic-independent genetic maintenance directly into their design, thereby supporting predictable manufacturing, environmental safety, and future clinical translation.

Concurrently, chromosomal integration of functional genes is favoured over episomal plasmid systems. Gene expression can be achieved via episomal plasmids or chromosomal integration, each with distinct regulatory and functional implications. Episomal deployment facilitates higher gene copy numbers and greater protein production [24,25,26] but plasmid maintenance imposes metabolic burden, increases instability, and raises concerns regarding horizontal gene transfer and dissemination of antibiotic resistance determinants [27,28,29]. In contrast, chromosomal strain offers superior genetic stability and eliminates plasmid-associated risks, albeit typically with lower expression due to reduced copy number [30,31]. For LBPs intended for clinical translation, integrated systems therefore represent a balanced approach between functionality and regulatory acceptability [3].

A widely adopted strategy to mitigate these biosafety risks is the engineering of microbial auxotrophy, whereby cells depend on externally supplied metabolites for growth [19]. Disruption of genes involved in essential biosynthetic pathways generates strains that survive only in supplemented environments, thereby reducing ecological fitness and limiting persistence outside controlled settings [3]. As an intrinsic biocontainment strategy, auxotrophy is well aligned with the environmental protection principles established under EU contained-use legislation [20]. In the present study, disruption of the *ilvC* gene was selected to implement this strategy. In *Escherichia coli*, *ilvC* encodes ketol-acid reductoisomerase, an essential enzyme in the biosynthesis of the branched-chain amino acids isoleucine and valine. Deletion of *ilvC* generates a stable auxotrophic phenotype that restricts bacterial proliferation in the absence of external supplementation while preserving viability under controlled culture conditions [24,32,33]. This genetically encoded containment mechanism therefore provides intrinsic biosafety without requiring external containment systems or antibiotic-dependent genetic maintenance.

**Figure 1:**
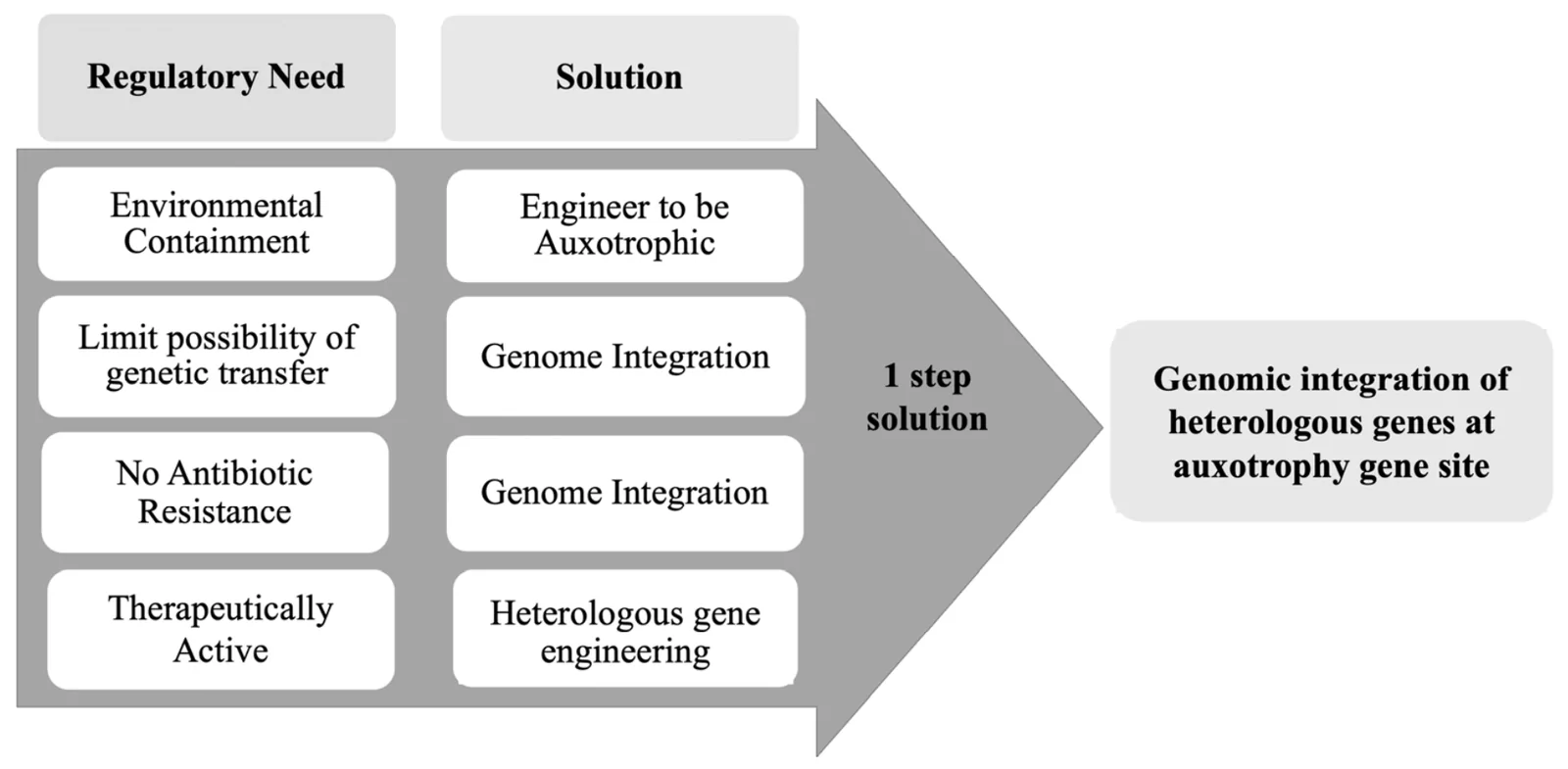
Conceptual overview of the single-step chromosomal engineering strategy proposed to generate a regulatory-oriented live biotherapeutic chassis. By integrating auxotrophy-mediated biocontainment with site-specific chromosomal insertion of a therapeutic heterologous gene into the auxotrophy locus, the strategy simultaneously addresses environmental containment, elimination of antibiotic resistance markers, stable therapeutic gene maintenance, and preservation of therapeutic functionality. This integrated strategy provides a streamlined framework for developing genetically stable, intrinsically biosafe, and regulatory-oriented live biotherapeutic chassis.

Conventional engineering of live biotherapeutic strains frequently relies on multi-step plasmid-based workflows involving iterative cloning, transformation, and antibiotic selection while maintaining multicopy replicons that impose metabolic burden, reduce genetic stability, and increase opportunities for horizontal gene transfer [12,15,27]. These limitations have stimulated growing interest in chromosomal engineering strategies that eliminate plasmid dependence while improving genetic stability, simplifying strain construction, and facilitating alignment with current biosafety principles for contained-use applications and supporting future translational development [12,13]. Figure 1 summarises the engineering rationale adopted in this study, illustrating how a single-step chromosomal integration strategy can simultaneously address key regulatory and biosafety requirements while preserving therapeutic functionality.

While auxotrophy addresses biological containment, successful bacterial DNA delivery also requires efficient intracellular release of the therapeutic payload. To preserve this functionality, the *hlyA* gene, encoding Listeriolysin O (LLO) from *Listeria monocytogenes*, was selected because of its well-established ability to disrupt endosomal membranes following bacterial internalisation. Controlled LLO expression facilitates endosomal escape, enhancing cytosolic delivery of therapeutic cargo and improving the efficiency of bacterial-mediated DNA transfer. Accordingly, LLO has been extensively employed in engineered microbial systems for intracellular delivery, antigen presentation, and vaccine applications [34,35,36,37,38].

These complementary engineering strategies were implemented in *E. coli* MG1655, a non-pathogenic K-12 derivative widely recognised as a genetically tractable and biosafe bacterial chassis. MG1655 provides a well-characterised genomic background for rational redesign and lacks the virulence-associated features that would complicate its use as an experimental live biotherapeutic platform [39,40]. MG1655 also exhibits predictable metabolic behaviour and a well-characterised stress response network, facilitating rational genome engineering while minimising unintended pleiotropic effects. Furthermore, K-12 derivatives have been widely used in engineered microbial therapeutics and have demonstrated favourable safety profiles and translational potential [12,13,15,16]. These attributes make MG1655 an appropriate chassis for developing genetically stable, intrinsically contained, and therapeutically functional live biotherapeutic platforms.

To strengthen regulatory acceptability, all genetic modifications were introduced using the no-SCAR genome engineering platform, which combines CRISPR/Cas9 with λ-Red recombineering to generate seamless, scarless chromosomal edits without permanent antibiotic resistance markers [41,42]. This strategy is consistent with current regulatory expectations established by the European Medicines Agency (EMA), the U.S. Food and Drug Administration (FDA), and the International Council for Harmonisation (ICH), which emphasise genetic stability, minimisation of antimicrobial resistance markers, biosafety, and robust quality control throughout the development of live biotherapeutic products. Likewise, it is aligned with the principles of EU Directive 2009/41/EC governing the contained use of genetically modified microorganisms and supports the biosafety requirements applied by national competent authorities, including the Environmental Protection Agency (EPA) and the Health Products Regulatory Authority (HPRA) in Ireland. By eliminating permanent selection markers and integrating the therapeutic cassette directly into the chromosome, this regulatory-oriented engineering strategy reduces genetic complexity, minimises the potential for horizontal gene transfer, and provides a genetically stable, plasmid-free chassis for future translational development. The chromosomally integrated *hlyA* construct was subsequently compared with an episomal (plasmid-based) counterpart to evaluate differences in genetic stability, expression, and functional activity.

Accordingly, this study aimed to establish a proof-of-concept single-step chromosomal engineering strategy in *E. coli* MG1655 in which replacement of *ilvC* with an *hlyA* cassette simultaneously generates an auxotrophic biocontainment phenotype and a chromosomally encoded therapeutic delivery function. We further compared this integrated chromosomal design with an episomal *hlyA* system to examine growth, haemolytic activity, plasmid-retention behaviour, structural maintenance of the engineered locus after serial passaging, and preliminary intracellular DNA-delivery performance.

## 2. Materials and Methods

### 2.1. Construction and Validation of the Chromosomal ΔilvC::hlyA Strain

Chromosomal integration of *hlyA* into the *ilvC* locus of *Escherichia coli* K-12 MG1655 (ATCC 700926) was achieved using the no-SCAR scarless genome-editing system, which combines λ-Red recombineering with CRISPR/Cas9-mediated counterselection [41]. Additional methodological details of the no-SCAR genome-editing strategy are provided in Supplementary Method S1. The *ilvC* coding sequence was replaced in a single homologous recombination event with an *hlyA* expression cassette flanked by approximately 1000 bp homology arms corresponding to the upstream and downstream chromosomal regions. Chemically competent MG1655 cells carrying the pCas9 and pKDsgRNA plasmids were prepared according to established no-SCAR protocols. Cells were transformed with the donor DNA and subjected to CRISPR/Cas9-mediated counterselection to enrich for correctly edited recombinants. Additional information regarding donor construct design, primer sequences, and the complete donor DNA sequence is provided in Supplementary Materials. Following genome editing, recombinant colonies were screened by PCR, and the genome-editing plasmids were cured by serial passaging at 37 °C under non-selective conditions, as described in Supplementary Method S4.

Correct chromosomal integration was validated using junction-spanning PCR with primer pairs designed to amplify both upstream and downstream insertion boundaries. PCR reactions were performed using Q5 High-Fidelity DNA Polymerase (New England Biolabs, Ipswich, MA, USA), and amplicons were analysed by agarose gel electrophoresis to verify the expected fragment sizes. Selected PCR products were purified and submitted for Sanger sequencing (Eurofins Genomics, Ebersberg, Germany) using external and internal primers. Sequence alignments were performed against the donor construct and the *E. coli* MG1655 reference genome using SnapGene, Version 8.0.1 (Dotmatics, San Diego, CA, USA) to confirm precise chromosomal integration without unintended insertions, deletions, or sequence rearrangements. Additional details of the PCR verification strategy are provided in Supplementary Method S3, and representative PCR products together with primer positions are presented in Supplementary Figure S4.

Functional disruption of *ilvC* was verified by auxotrophy testing in M9 minimal medium supplemented with or without isoleucine and valine (50 mg/L each). Growth of the engineered strain was observed exclusively in medium supplemented with both amino acids, confirming successful disruption of the branched-chain amino acid biosynthetic pathway following *ilvC* deletion. Additional experimental details of the auxotrophy assay, including culture conditions and medium composition, are provided in Supplementary Method S5.

### 2.2. Development and Validation of Episomal Expression Systems

The episomal comparator consisted of the pNZ44-*hlyA* plasmid, carrying the *L. monocytogenes hlyA* coding sequence (GenBank accession M24199.1) [43], and the GFP reporter plasmid pMAX-GFP (Addgene, Watertown, MA, USA; plasmid #177825). The former was used for LLO expression, whereas the latter served as a reporter for plasmid-retention and intracellular DNA delivery during bactofection assays. Chemically competent *E. coli* MG1655 cells were prepared using a calcium chloride protocol. Briefly, mid-logarithmic-phase cultures were harvested by centrifugation, washed with ice-cold CaCl_2_ solution, and resuspended in 0.1 M CaCl_2_ containing 15–20% glycerol. Competent cells were used immediately or stored at −80 °C until required. For plasmid transformation, 50 µL of competent cells were mixed with 1–5 µL plasmid DNA, incubated on ice for 30 min, heat-shocked at 42 °C for 45 s, and returned to ice for 2 min. Cells were recovered in SOC medium for 45–60 min at 37 °C before plating on selective LB agar. Single-plasmid transformants were selected on LB agar containing chloramphenicol (25 µg/mL) for pNZ44-*hlyA* or kanamycin (50 µg/mL) for pMAX-GFP.

Following molecular confirmation, single-plasmid transformants were rendered chemically competent again and transformed with the complementary plasmid to generate dual-plasmid strains. Co-transformants were selected on LB agar supplemented with both antibiotics and re-streaked to confirm stable plasmid maintenance. Successful plasmid transformation was confirmed by colony PCR using *hlyA*- and *gfp*-specific primers, followed by agarose gel electrophoresis to confirm expected amplicon sizes. Plasmid identity and structural integrity were further confirmed by plasmid extraction and diagnostic restriction digestion.

### 2.3. Growth Kinetics of Chromosomal and Episomal Strains

Growth kinetics were evaluated to determine whether chromosomal integration of *hlyA*, deletion of *ilvC*, or episomal plasmid carriage affected bacterial fitness under the experimental conditions. Overnight cultures were diluted to an initial OD_600_ of 0.05 in fresh LB broth and incubated in 10 mL cultures in 50 mL Falcon tubes at 37 °C with shaking at 227 rpm. Optical density at 600 nm was measured hourly for 19 h using an ODX spectrophotometer [44]. Culture medium without bacterial inoculum was included as a blank control for background correction. Growth kinetics were evaluated as exploratory proof-of-concept experiments using single biological cultures and are therefore presented descriptively, without inferential statistical analysis.

### 2.4. Comparative Haemolytic Activity of Chromosomal and Episomal hlyA Expression Systems

Haemolytic activity was evaluated to compare the functional activity of LLO produced from chromosomal and episomal *hlyA* expression systems. Columbia agar supplemented with 5% defibrinated sheep blood (Oxoid, Thermo Fisher Scientific, Basingstoke, UK) was prepared according to established haemolysis protocols [45]. Strains expressing *hlyA* either episomally (pNZ44-*hlyA*) or chromosomally (Δ*ilvC*::*hlyA*) were streaked onto blood agar plates alongside *L. monocytogenes* as a positive control and non-haemolytic *E. coli* MG1655 as a negative control. Plates were incubated aerobically at 37 °C for 24–72 h. Following incubation, haemolytic activity was assessed qualitatively based on the presence and type of haemolytic zone surrounding bacterial colonies and classified as β-haemolysis (complete erythrocyte lysis), α-haemolysis (partial haemolysis), or γ-haemolysis (absence of haemolysis) according to established criteria [45]. Representative images were recorded photographically. The assay was performed independently on three occasions to confirm reproducibility of the observed haemolytic phenotype. Because haemolysis was assessed qualitatively and haemolytic zones were not quantitatively measured, no inferential statistical analyses were performed.

### 2.5. Plasmid Stability Analysis

Plasmid stability was evaluated to investigate whether chromosomal integration of the LLO expression cassette improved maintenance of a therapeutic reporter plasmid during serial propagation in the absence of antibiotic selection. Wild-type MG1655, chromosomal and episomal MG1655 strains carrying the GFP reporter plasmid pMAX-GFP were included in the analysis. In this experimental model, pMAX-GFP served as a surrogate therapeutic plasmid to evaluate plasmid retention in bacterial chassis differing in LLO delivery strategy. Overnight cultures grown under antibiotic selection were diluted 1:100 into fresh antibiotic-free LB medium and incubated at 37 °C with shaking. Serial passaging was performed every 12 h by transferring 1% (*v*/*v*) of each culture into fresh antibiotic-free LB medium. Ten consecutive passages, corresponding to approximately 100 bacterial generations, were completed. At generations 0, 20, 40, 60, 80, and 100, aliquots were plated onto selective and non-selective LB agar. Plasmid retention was calculated as the percentage of colonies recovered on selective LB agar relative to the total number of colonies recovered on non-selective LB agar and classified as very stable (>90%), stable (70–90%), moderately stable (40–70%), unstable (10–40%), or highly unstable (<10%) [46,47,48,49,50]. For each passage, three parallel cultures originating from the same overnight culture were analysed, and plasmid retention was calculated from the mean colony counts obtained under selective and non-selective conditions.

### 2.6. Chromosomal Integration Stability Following Serial Passaging

To validate chromosomal integration of *hlyA* at the *ilvC* locus and assess its genetic stability after serial passaging, the engineered *E. coli* MG1655 Δ*ilvC*::*hlyA* strain was analysed by PCR and Sanger sequencing. To test stability in the absence of antibiotic selection, the strain was serially passaged under non-selective conditions as described in Section 2.5. After approximately 100 bacterial generations, individual colonies were isolated for molecular analysis.

Genomic DNA was extracted from selected colonies, and chromosomal integration was evaluated using two complementary PCR strategies. First, primers flanking the integration site were used to amplify an amplicon spanning the integrated donor region, generating a product of approximately 4.1 kb encompassing the *hlyA* insertion and adjacent donor-derived sequences. Second, combinations of integration-specific and internal *hlyA* primers were used to confirm the integrity of the 5′ and 3′ donor-side junctions and to provide sequence coverage across the insert. PCR products were initially analysed by agarose gel electrophoresis, and selected amplicons were subsequently subjected to Sanger sequencing.

For sequence validation, individual Sanger reads were aligned to the donor reference sequence, which comprised a 5′ homology arm (1000 bp), the *hlyA* coding sequence (1590 bp), and the downstream donor region including the 3′ homology arm. Read orientation was assigned according to the highest-scoring alignment to the forward or reverse-complement reference orientation. The resulting alignments were used to assess read coverage across the 5′ homology arm, the 5′ arm–*hlyA* junction, internal *hlyA* regions, the distal *hlyA* region, and the downstream donor-side sequence including the 3′ homology arm. Integration was considered supported when sequencing reads confirmed continuity between donor-derived flanking regions and the *hlyA* insert at the expected chromosomal junctions. Because sequence coverage across *hlyA* was assembled from multiple partially overlapping reads, sequence integrity was interpreted conservatively, distinguishing strong support for insert presence and junction preservation from complete sequence-level validation of the entire coding sequence. The primers used for PCR amplification and Sanger sequencing are listed in Table 1.

### 2.7. Comparative Evaluation of Chromosomal and Episomal DNA Delivery in RAW 264.7 Macrophages

Bactofection assays were performed to compare the intracellular DNA delivery efficiency of chromosomal and episomal LLO-based bacterial delivery systems in RAW 264.7 macrophages. RAW 264.7 murine macrophages were maintained in Dulbecco’s Modified Eagle Medium (DMEM) supplemented with 10% foetal bovine serum (FBS), 100 U/mL penicillin, and 100 μg/mL streptomycin at 37 °C in a humidified atmosphere containing 5% CO_2_. For bactofection assays, cells were seeded in 24-well plates at 5 × 10^5^ cells/mL and allowed to adhere overnight. Chromosomal Δ*ilvC*::*hlyA* and episomal *hlyA*-expressing MG1655 strains, each carrying the GFP reporter plasmid pMAX-GFP, were used as bacterial delivery systems. The GFP reporter plasmid served as a surrogate therapeutic cargo for evaluating intracellular plasmid delivery following bacterial escape from the phagosome. Bacteria were grown overnight in LB medium containing the appropriate antibiotics, diluted 1:20 into fresh LB medium, and cultured to mid-logarithmic phase (OD_600_ = 0.4). A previously established OD_600_–CFU calibration curve generated under identical culture conditions was used to estimate bacterial inocula. Multiplicity of infection (MOI) was calculated according to Byrne et al. (2014) [43]. For wells containing 5 × 10^5^ macrophages, MOIs of 300 and 500 corresponded to approximately 1.5 × 10^8^ and 2.5 × 10^8^ CFU per well, respectively. Macrophages were infected at MOI 300 or MOI 500. Plates were centrifuged at 500× *g* for 5 min to promote bacteria–cell contact and incubated for 1 h at 37 °C in 5% CO_2_. Cells were then washed three times with PBS and cultured in DMEM supplemented with antibiotics for an additional 24 h to allow intracellular GFP expression. GFP expression was evaluated by fluorescence microscopy and flow cytometry. A minimum of 10,000 viable events was acquired per sample. Viable cells were identified using forward- and side-scatter parameters, doublets were excluded, and the percentage of GFP-positive cells was quantified using BD FACSDiva™ software (BD Biosciences, Franklin Lakes, NJ, USA) and analyzed using FlowJo™ software, Version 10 (BD Biosciences, Ashland, OR, USA). These proof-of-concept bactofection experiments were performed without biological replication. Consequently, the results are presented descriptively and should be interpreted as preliminary comparative evidence of platform performance rather than statistically validated quantitative measurements.

### 2.8. Experimental Design and Data Analysis

The experimental design was tailored to the objective of each assay. Construction and validation of the engineered strain, including PCR confirmation, sequencing, auxotrophy assessment, growth kinetics, haemolytic activity, plasmid-retention analysis, and bactofection experiments, were conducted as exploratory studies to evaluate the feasibility and functional performance of the engineered bacterial chassis. Replication was implemented according to the purpose of each assay. The haemolytic activity assay was repeated on three separate occasions to confirm reproducibility of the observed phenotype. Plasmid-retention analysis was performed using three parallel cultures derived from the same overnight culture, and plasmid retention was calculated from the mean colony counts obtained under selective and non-selective conditions. Because the primary objective of these experiments was methodological validation rather than hypothesis testing, results are presented descriptively, and no inferential statistical analyses were performed. GraphPad Prism v10 (GraphPad Software, San Diego, CA, USA) was used for data organisation and graphical representation, where applicable.

## 3. Results

### 3.1. Construction and Molecular Validation of the Chromosomal ΔilvC::hlyA Strain

**Figure 2:**
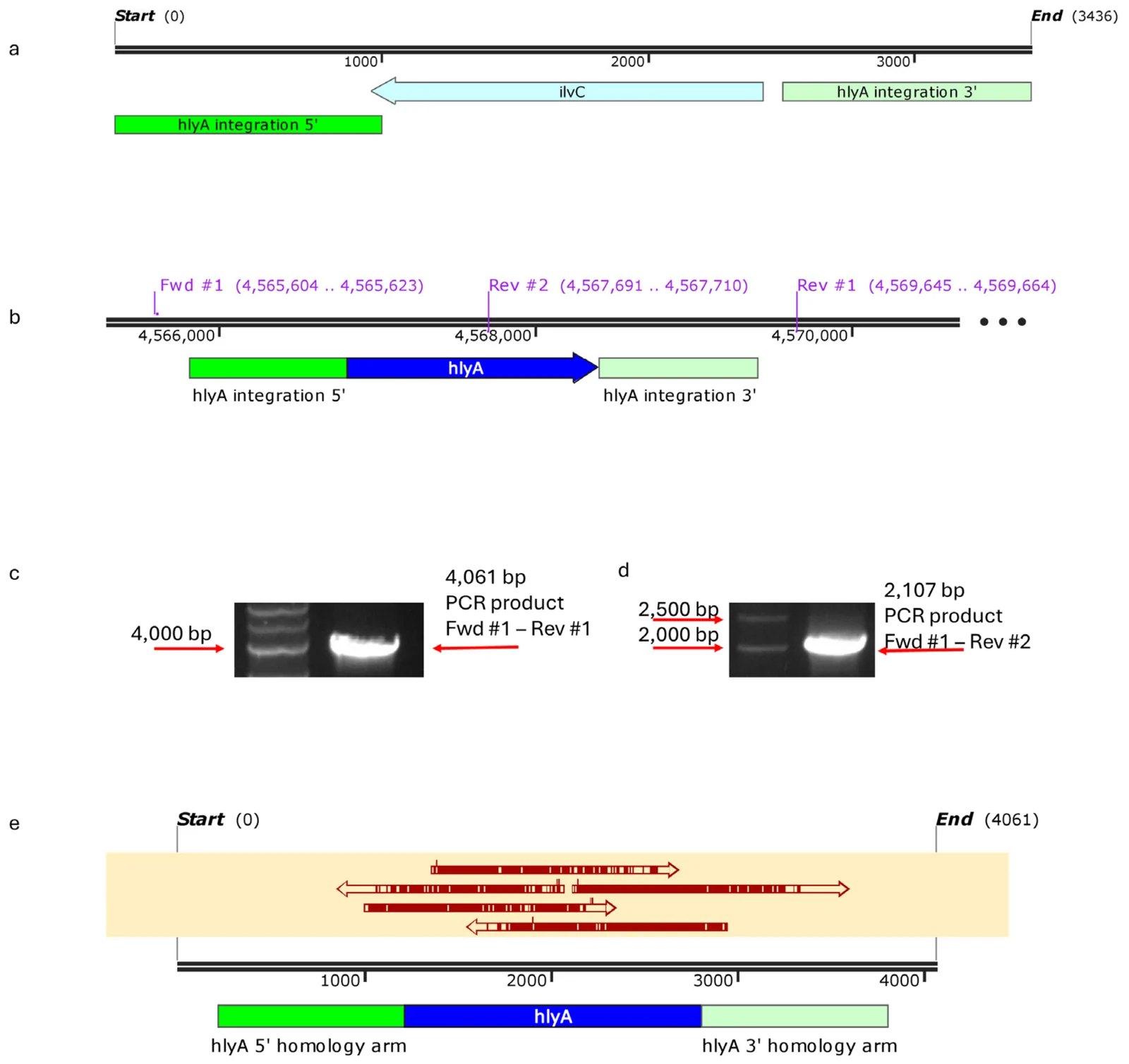
Construction and molecular validation of the chromosomal Δ*ilvC*::*hlyA* strain. (**a**) Schematic representation of the donor construct used for CRISPR/Cas9-mediated no-SCAR genome editing, showing the 5′ and 3′ homology arms flanking the *hlyA* expression cassette for replacement of the *ilvC* coding sequence. (**b**) Schematic representation of the edited chromosomal locus following homologous recombination, indicating the integrated *hlyA* cassette and the positions of the primers used for molecular validation. (**c**) Junction-spanning PCR amplification using primers Fwd #1 and Rev #1, generating the expected 4061 bp product and confirming correct chromosomal integration of the *hlyA* cassette. (**d**) PCR amplification using primers Fwd #1 and Rev #2, generating the expected 2107 bp product and providing additional confirmation of correct integration at the edited locus. (**e**) Schematic representation of the Sanger sequencing reads aligned across the integrated donor construct, illustrating sequence coverage spanning the 5′ homology arm, the *hlyA* coding sequence, and the 3′ homology arm to confirm accurate chromosomal integration. Red arrows represent the individual sequencing reads obtained using five primers, with complete overlapping coverage across the edited locus. Solid red lines within the arrows indicate aligned sequence, whereas gaps indicate sequence mismatches. Colours distinguish the different genetic elements shown in the schematic.

Successful chromosomal integration of the *hlyA* expression cassette into the *ilvC* locus of *E. coli* MG1655 was achieved using the CRISPR/Cas9-mediated no-SCAR genome-editing strategy (Figure 2). The donor DNA was inserted into the target locus by homologous recombination, thereby generating a chromosomal *hlyA* expression cassette together with the intended Δ*ilvC* auxotrophic genotype. Correct genomic integration and sequence integrity were confirmed by junction-spanning PCR and Sanger sequencing. PCR amplification generated products of the expected sizes for both junction-spanning and internal verification assays, and Sanger sequencing confirmed precise insertion of the *hlyA* cassette without unintended sequence alterations (Supplementary Figure S4). Following genome editing, the editing plasmids were successfully cured through serial passaging under non-selective conditions, as described in Supplementary Method S4.

**Figure 3:**
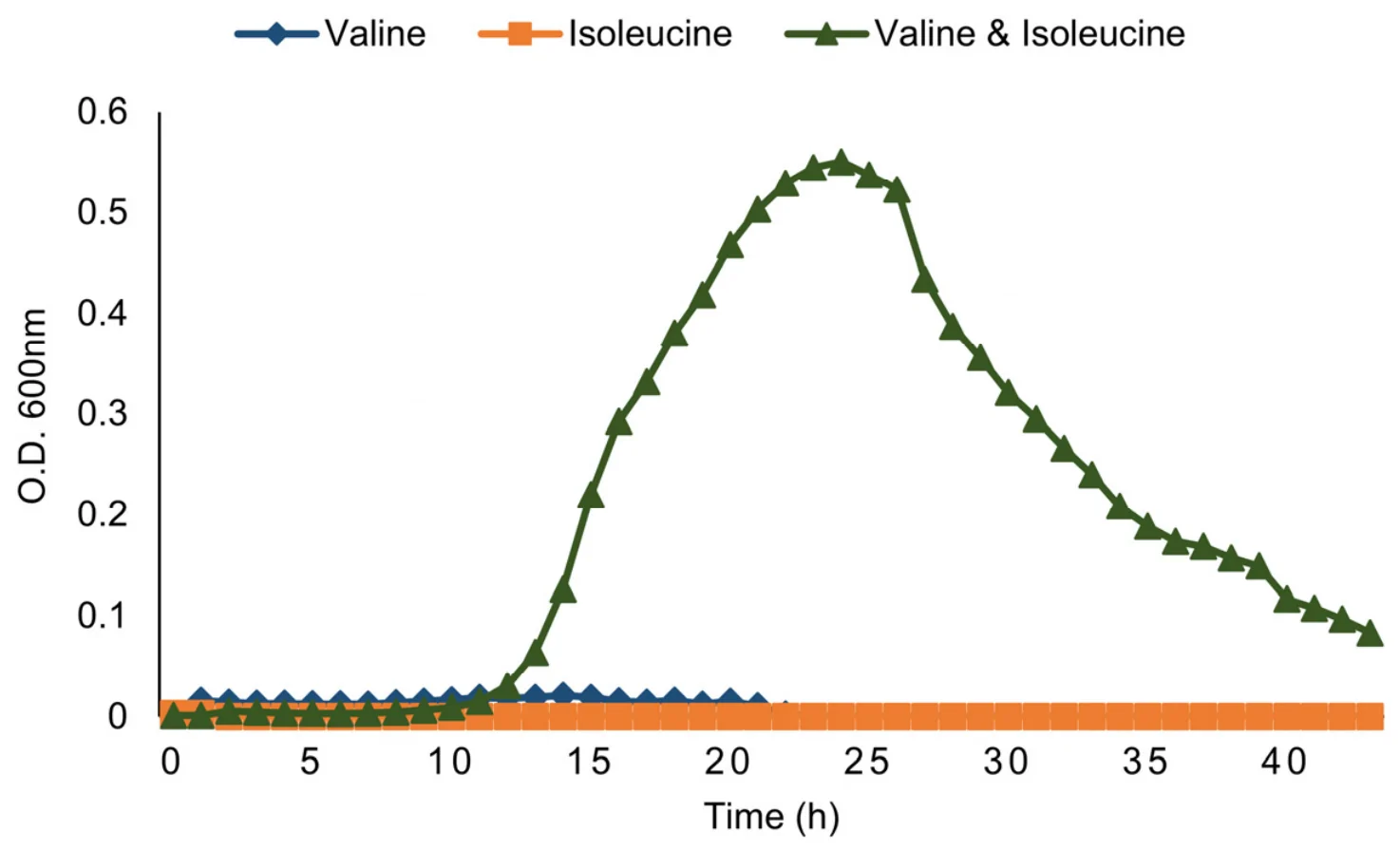
Functional validation of the Δ*ilvC* auxotrophic phenotype. Growth of the engineered *E. coli* MG1655 Δ*ilvC*::*hlyA* strain in M9 minimal medium in the absence or presence of branched-chain amino acid supplementation. The engineered strain failed to grow in unsupplemented medium but recovered growth following supplementation with isoleucine and valine, confirming functional disruption of the *ilvC* biosynthetic pathway and establishment of the intended auxotrophic phenotype.

Functional validation of the Δ*ilvC* genotype was performed by auxotrophy testing. The engineered strain failed to grow in M9 minimal medium lacking branched-chain amino acid supplementation, whereas growth was restored following supplementation with isoleucine and valine (Figure 3), confirming successful disruption of the *ilvC* biosynthetic pathway and establishment of the intended auxotrophic phenotype.

### 3.2. Growth Kinetics of Engineered E. coli Strains

Growth kinetics were evaluated to compare the effects of chromosomal *hlyA* integration and episomal *hlyA* expression on bacterial growth (Supplementary Figure S3). Because growth analysis was performed using single biological cultures, the results are presented descriptively. The parental MG1655 strain and the chromosomal Δ*ilvC*::*hlyA* strain exhibited highly similar growth kinetics throughout cultivation, reaching stationary phase after approximately 12 h. The chromosomal strain displayed a slightly prolonged exponential phase and attained a marginally higher final optical density than the parental strain. In contrast, the episomal strain carrying pNZ44-*hlyA* exhibited delayed exponential growth and a lower final optical density throughout the experiment. These observations indicate that chromosomal *hlyA* integration had minimal impact on bacterial growth under the conditions tested, whereas the episomal *hlyA* expression system was associated with slower growth and a lower final optical density.

### 3.3. Comparative Haemolysis Analysis of Chromosomal and Episomal LLO Expression

Functional expression of Listeriolysin O (LLO) was evaluated using Columbia sheep blood agar to determine whether chromosomal integration of *hlyA* preserved haemolytic activity. As expected, the parental MG1655 strain lacking *hlyA* showed no detectable haemolysis, whereas *L. monocytogenes* (positive control) displayed the characteristic β-haemolytic phenotype. Both the chromosomal Δ*ilvC*::*hlyA* strain and the episomal *hlyA*-expressing strain (MG1655 [pNZ44-*hlyA*]) produced distinct β-haemolytic zones, demonstrating functional expression of LLO. No obvious qualitative differences in the haemolytic phenotype were observed between the chromosomal and episomal expression systems under the conditions tested. The assay was repeated on three separate occasions, yielding reproducible haemolytic phenotypes across all experiments. Representative haemolysis images are provided in the Supplementary Materials (Figure S2).

### 3.4. Comparative Plasmid Retention of Chromosomal and Episomal Strains

**Figure 4:**
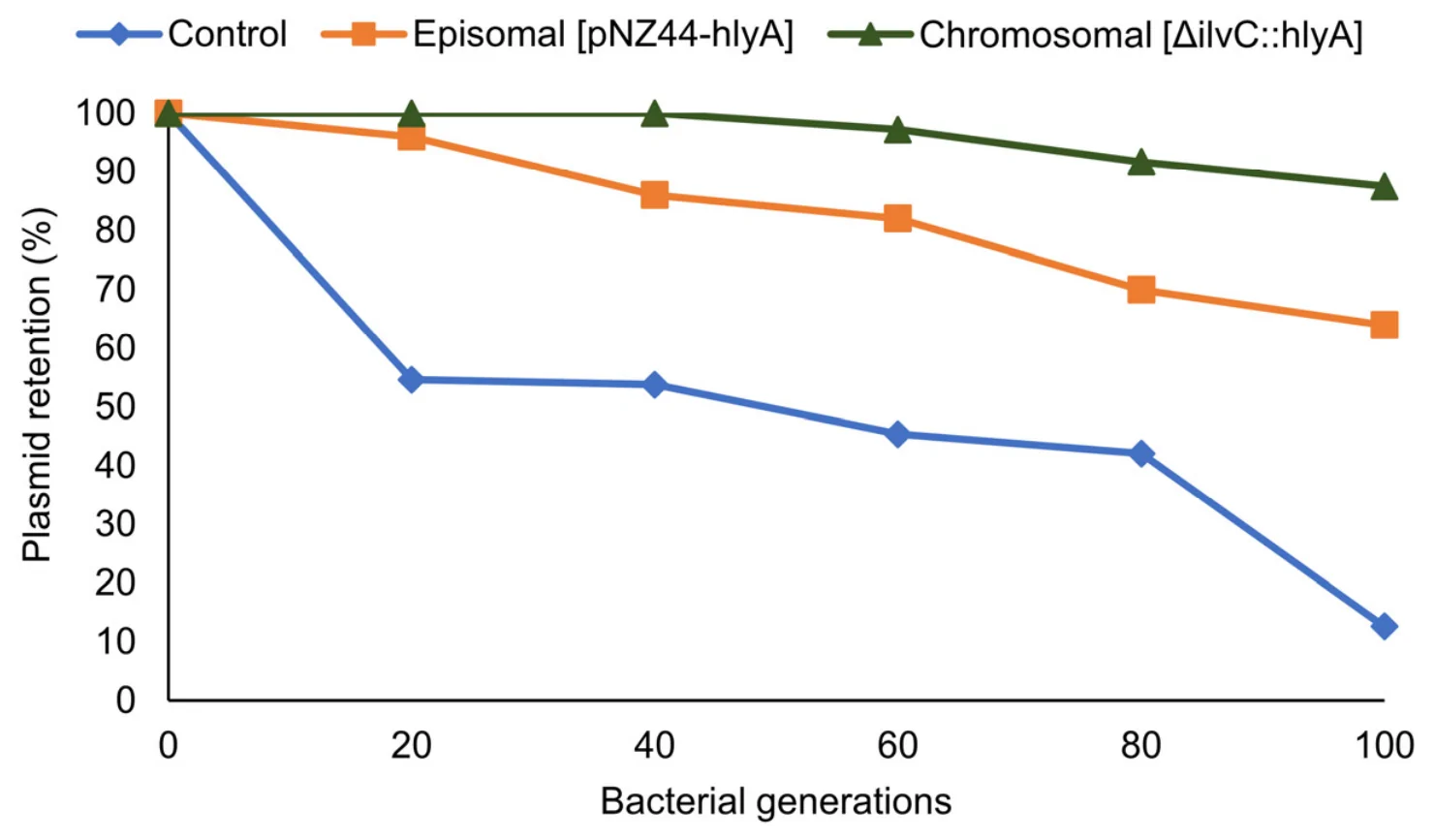
Comparative plasmid retention profiles of control, episomal, and chromosomal *E. coli* MG1655 strains during serial passaging under non-selective conditions. Plasmid retention was determined by monitoring maintenance of the pMAX-GFP reporter plasmid and is expressed as the percentage of pMAX-GFP-positive colonies relative to generation 0 (100%), as determined by colony recovery on selective medium. The control strain (MG1655 [pMAX-GFP]), the episomal strain (MG1655 [pNZ44-*hlyA*, pMAX-GFP]), and the chromosomal strain (MG1655 Δ*ilvC::hlyA* [pMAX-GFP]) were monitored over 100 bacterial generations to evaluate long-term plasmid stability.

Validation of the episomal expression system is presented in Supplementary Figure S1 and served as the basis for subsequent comparative analyses. Distinct plasmid-retention profiles were observed following serial passaging under non-selective conditions (Figure 4). The control strain carrying pMAX-GFP alone exhibited progressive plasmid loss, with retention decreasing from 100% at baseline to approximately 12% after 100 bacterial generations. The episomal strain carrying pNZ44-*hlyA* showed improved plasmid maintenance, retaining approximately 64% of pMAX-GFP-positive colonies after 100 generations. In contrast, the chromosomal strain showed the highest plasmid stability throughout the experiment. Plasmid retention remained at 100% through generation 40, exceeded 95% through generation 60, and was still 88% after approximately 100 generations. Collectively, these observations indicate that chromosomal integration of the therapeutic module was associated with improved plasmid retention during prolonged cultivation in the absence of antibiotic selection, thereby reducing dependence on continuous selective pressure for maintenance of plasmid-borne genetic cargo.

### 3.5. Structural Maintenance of the Chromosomal ΔilvC::hlyA Locus After Serial Passaging

**Figure 5:**
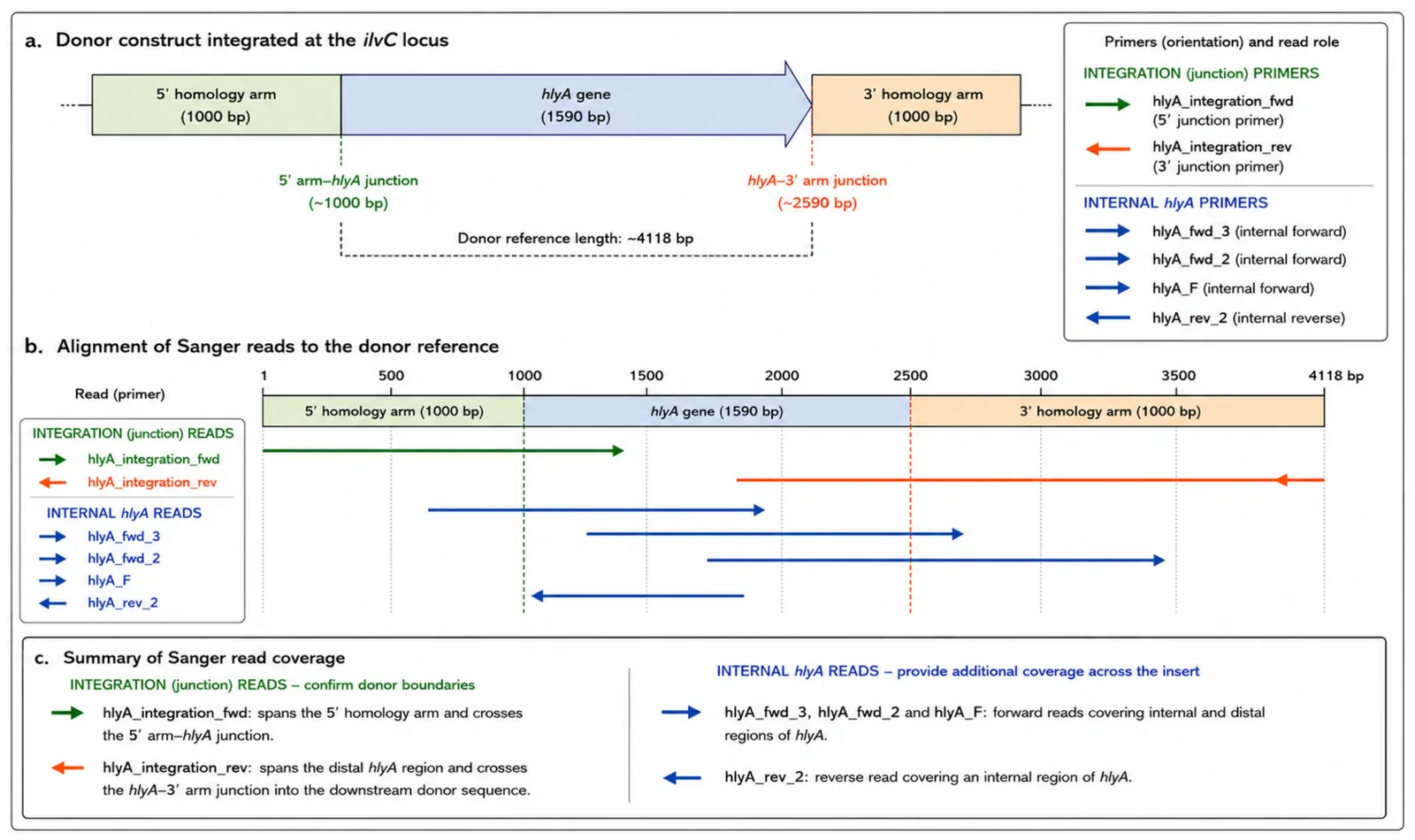
Sanger sequencing support for structural maintenance of the chromosomal Δ*ilvC*::*hlyA* locus after serial passaging. (**a**) Schematic representation of the donor construct integrated at the *ilvC* locus, comprising the 5′ homology arm, the *hlyA* coding sequence, and the 3′ homology arm. Dashed lines indicate the expected 5′ arm–*hlyA* and *hlyA*–3′ arm junctions, which were targeted for sequencing confirmation. (**b**) Alignment of Sanger sequencing reads obtained from post-passaging Δ*ilvC*::*hlyA* colonies to the donor reference sequence. Green arrows represent the forward integration-specific read spanning the 5′ arm–*hlyA* junction (hlyA_integration_fwd), orange arrows represent the reverse integration-specific read spanning the *hlyA*–3′ arm junction (hlyA_integration_rev), and blue arrows represent internal *hlyA* sequencing reads (hlyA_fwd_3, hlyA_fwd_2, hlyA_F, and hlyA_rev_2). Integration-specific reads (hlyA_integration_fwd and hlyA_integration_rev) span the 5′ and 3′ junctions, respectively, confirming preservation of the donor boundaries, while internal *hlyA* sequencing reads provide supplementary coverage across the coding sequence. (**c**) Summary of Sanger sequencing coverage across the integrated donor construct. Integration-specific reads confirm the integrity of the 5′ and 3′ junctions, whereas internal *hlyA* reads provide overlapping coverage of the coding sequence, supporting structural maintenance of the engineered chromosomal Δ*ilvC*::*hlyA* locus after prolonged non-selective propagation.

To determine whether the engineered chromosomal locus remained structurally intact after prolonged propagation in the absence of antibiotic selection, PCR amplicons generated from post-passaging Δ*ilvC*::*hlyA* colonies were analysed by Sanger sequencing using six reads positioned across the donor-derived region (Figure 5). Reads were aligned to the donor reference sequence comprising the 5′ homology arm, the *hlyA* coding sequence, and the downstream donor-side region including the 3′ homology arm.

The integration-specific reads provided the strongest evidence for preservation of the edited locus after serial passaging. Read *hlyA*_integration_fwd mapped across the upstream donor region and extended through the 5′ homology arm–*hlyA* junction, whereas *hlyA*_integration_rev mapped to the distal portion of *hlyA* and extended into the downstream donor-side sequence, supporting continuity across the *hlyA*–3′ donor-side junction. Additional internal reads aligned to proximal, internal, and distal regions of *hlyA*, providing supplementary coverage across the insert. Collectively, these sequencing data were consistent with structural maintenance of the Δ*ilvC*::*hlyA* locus at the expected chromosomal site after approximately 100 bacterial generations under non-selective conditions.

Additional internal reads further supported the presence of the insert. Read hlyA_fwd_3 mapped to the proximal/internal region of *hlyA*, whereas hlyAF, hlyA_fwd_2, and hlyA_rev_2 aligned to internal and distal regions of the coding sequence, providing supplementary coverage across the insert. Together, these data support the presence of *hlyA* between the expected donor flanking regions and are consistent with correct chromosomal integration of the donor cassette at the *ilvC* locus. A summary of the sequencing evidence supporting each region of the integrated donor cassette is provided in Table 2. Overall, the Sanger dataset provides strong support for correct donor integration, particularly for the 5′ junction, the distal *hlyA* region, and the downstream donor-side sequence defined by the integration-specific reads.

### 3.6. Comparative Evaluation of Chromosomal and Episomal DNA Delivery

**Figure 6:**
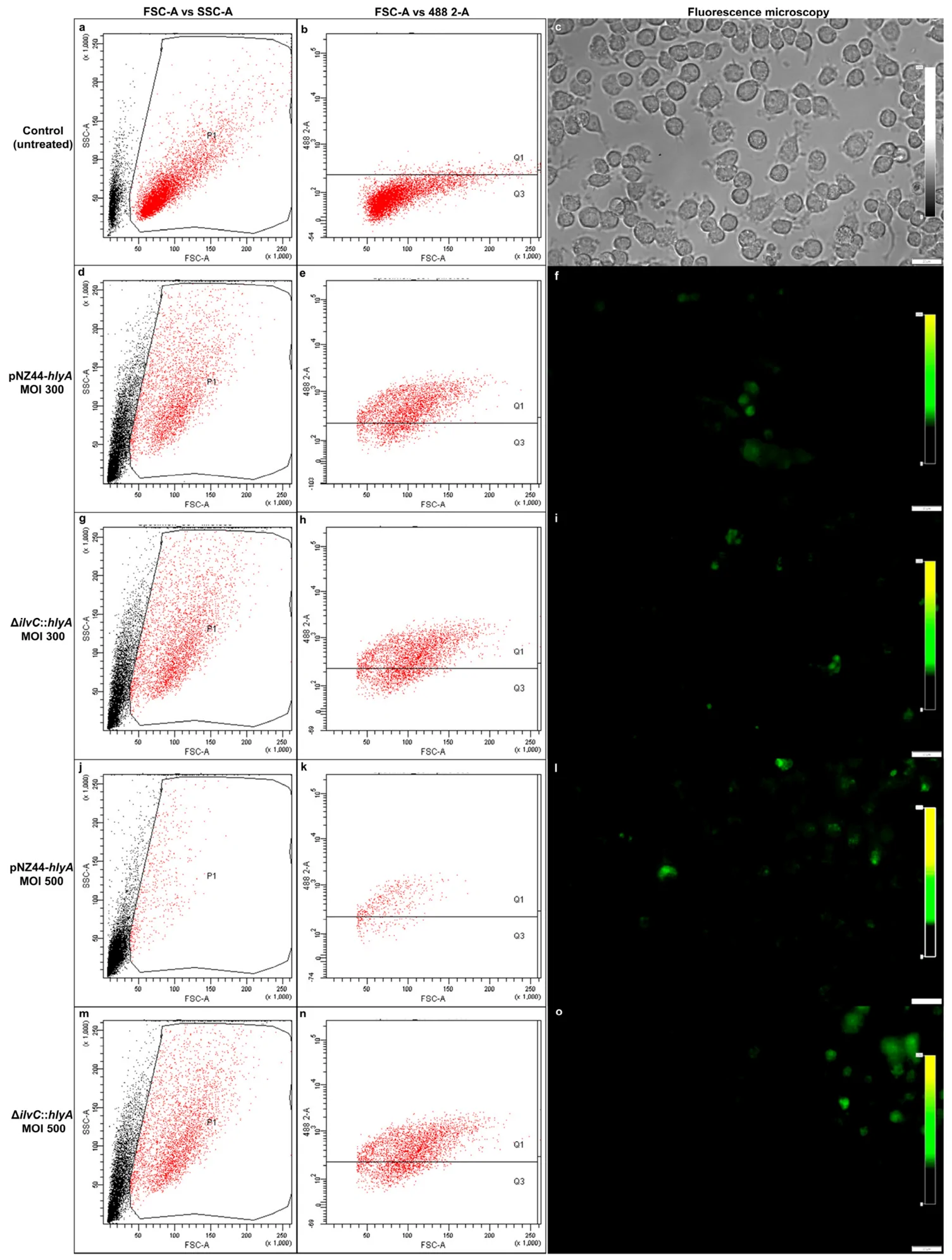
Representative flow-cytometry and fluorescence microscopy analyses of GFP expression in RAW 264.7 macrophages following bactofection. Flow-cytometry plots show cell gating (FSC-A versus SSC-A) and GFP fluorescence (FSC-A versus 488-2A), whereas microscopy images provide qualitative visualization of GFP-positive macrophages. In the flow-cytometry plots, black dots represent the total acquired events, red dots represent the gated macrophage population (P1), the polygon outlines the gating region, and the horizontal line in the FSC-A versus 488-2A plots indicates the threshold separating GFP-positive (Q1) from GFP-negative (Q3) events. In the microscopy panels, green fluorescence indicates GFP-expressing cells. (**a**–**c**) Untreated control cells. (**d**–**f**) Macrophages bactofected with the episomal strain (*pNZ44-hlyA* carrying pMAX-GFP) at MOI 300. (**g**–**i**) Macrophages bactofected with the chromosomal strain (Δ*ilvC*::*hlyA* carrying pMAX-GFP) at MOI 300. (**j**–**l**) Macrophages bactofected with the episomal strain at MOI 500. (**m**–**o**) Macrophages bactofected with the chromosomal strain at MOI 500. Images are representative of proof-of-concept experiments and are presented for qualitative comparison.

Because the bactofection experiments were performed as proof-of-concept studies without independent biological replication, flow-cytometry and fluorescence microscopy data are presented descriptively and should be interpreted as exploratory observations rather than statistically validated comparisons. Representative flow-cytometry analyses (Figure 6) showed detectable GFP-positive RAW 264.7 macrophages following bactofection with both the episomal (*pNZ44-hlyA*) and chromosomal (Δ*ilvC::hlyA*) delivery systems. Untreated control cells exhibited minimal GFP fluorescence, confirming the absence of detectable background reporter expression (Figure 6a–c).

At an MOI of 300, both delivery systems generated GFP-positive cell populations of similar magnitude within the gated macrophage population. Flow-cytometry measurements indicated 45.5% GFP-positive cells for the episomal strain and 42.6% for the chromosomal strain (Table 3). Cell viability was likewise similar between the two conditions (32.4% and 34.9%, respectively), supporting the qualitative observation that both delivery systems performed similarly under these experimental conditions. Fluorescence microscopy images qualitatively supported the flow-cytometry findings, revealing scattered GFP-positive macrophages following bactofection with both strains (Figure 6f,i).

Clear differences were observed at an MOI of 500. Exposure to the episomal strain resulted in a marked reduction in the gated macrophage population, accompanied by a decrease in cell viability to 4.9% and a reduction in GFP-positive cells to 10.7% (Table 3). Consistent with these observations, the corresponding FSC/SSC profile showed a substantially smaller gated cell population, while fluorescence microscopy revealed relatively few GFP-positive macrophages (Figure 6j–l). In contrast, the chromosomal strain retained a larger viable macrophage population at the same MOI, with cell viability of 23.0% while maintaining 37.5% GFP-positive cells (Table 3). The corresponding FSC/SSC profile showed a larger gated macrophage population than that observed for the episomal strain, consistent with greater preservation of host cells under high bacterial exposure. Fluorescence microscopy likewise demonstrated a greater number of GFP-positive macrophages with stronger overall fluorescence than observed for the episomal strain under the same conditions (Figure 6m–o).

Forward-scatter measurements were consistent with these observations. At an MOI of 500, macrophages exposed to the chromosomal strain exhibited a higher mean FSC-A value (68,533) than those exposed to the episomal strain (55,596), consistent with relatively greater preservation of cell size and structural integrity. Mean GFP fluorescence values were of similar magnitude across all bactofection conditions, indicating that the intensity of GFP expression among GFP-positive cells remained comparable despite differences in cell viability and the proportion of GFP-positive macrophages (Table 3).

The representative flow-cytometry and fluorescence microscopy observations indicate that chromosomal expression of *hlyA* was associated with sustained intracellular DNA delivery while preserving a larger viable macrophage population than the episomal system under the experimental conditions evaluated. These exploratory findings support the feasibility of chromosomal *hlyA* expression as a bactofection strategy, although confirmation will require independent biological replication and statistical analysis.

## 4. Discussion

This study provides proof-of-concept evidence that chromosomal integration represents a rational strategy for engineering a genetically stable, biosafe, and functionally reliable bacterial chassis for live biotherapeutic applications. By replacing plasmid-based *hlyA* expression with chromosomal integration at the *ilvC* locus, we established a simplified bacterial chassis in which therapeutic function and auxotrophy-mediated biocontainment are combined within a single genomic modification. Within the scope of this proof-of-concept study, the resulting strain preserved bacterial growth, retained LLO-associated haemolytic activity, showed favourable plasmid-retention and locus-maintenance characteristics during non-selective propagation, and remained capable of intracellular reporter-plasmid delivery in macrophages. These findings support the feasibility of the chromosomal design as an initial live biotherapeutic chassis framework [12,13,15].

A major challenge in the development of live biotherapeutic products (LBPs) is maintaining stable expression of therapeutic functions during manufacturing, storage, and in vivo application. Plasmid-based systems are particularly susceptible to segregational loss, copy-number variation, heterogeneous gene expression, and the metabolic burden associated with replication and maintenance of multicopy plasmids [12,13,15]. Chromosomal integration overcomes many of these limitations by coupling inheritance of the therapeutic cassette to chromosome replication, thereby reducing variability associated with plasmid maintenance while improving the long-term genetic predictability of the engineered strain. In addition, eliminating dependence on an extra expression plasmid reduces the requirement for antibiotic selection and decreases the potential for horizontal gene transfer, two important considerations for the development of clinically applicable bacterial therapeutics.

A distinctive feature of the platform described here is the implementation of a single genomic engineering event that simultaneously replaces the native *ilvC* locus with the *hlyA* expression cassette. Rather than introducing auxotrophy and therapeutic functionality as independent modifications, this strategy integrates both objectives into a single chromosomal redesign. Consequently, the engineered strain combines stable genomic expression of the therapeutic module with intrinsic metabolic containment while simplifying strain construction and reducing genetic complexity. This integrated design is conceptually consistent with current trends in synthetic biology, in which chassis engineering increasingly seeks to incorporate therapeutic performance, biosafety, manufacturability, and regulatory compatibility within a unified genetic framework [12,13].

Growth analyses further supported the advantages of chromosomal integration. Although the experiments were performed using a single biological sample, the episomal strain consistently exhibited a lower growth profile than both the parental and chromosomal strains. This qualitative observation is consistent with previous reports demonstrating that plasmid replication and constitutive expression impose an additional metabolic burden on bacterial cells by diverting cellular resources from growth and biomass production [27]. In contrast, chromosomal integration of *hlyA* produced a growth profile comparable to that of the parental MG1655 strain, indicating that stable genomic expression did not introduce an apparent fitness cost under the experimental conditions evaluated. These findings support the concept that chromosomal integration can preserve bacterial physiological performance while maintaining the engineered therapeutic function.

The selection of the *ilvC* locus was guided by both metabolic and biosafety considerations. The *ilvC* gene encodes ketol-acid reductoisomerase, an essential enzyme in the biosynthesis of the branched-chain amino acids isoleucine and valine. Deletion of this gene creates a strict auxotrophic phenotype that can be complemented only by external amino-acid supplementation, thereby establishing a predictable metabolic dependency. Such genetically encoded auxotrophies are widely recognised as one of the most robust intrinsic biocontainment strategies because they restrict bacterial persistence outside defined nutritional environments while avoiding the need for external containment mechanisms [15,16]. Consistent with this rationale, supplementation with branched-chain amino acids fully restored bacterial growth, demonstrating that the engineered auxotrophy imposed minimal physiological constraints under permissive conditions while maintaining effective metabolic containment.

Beyond establishing biological containment, replacement of *ilvC* with the *hlyA* expression cassette created a multifunctional chromosomal locus that simultaneously supports therapeutic-gene expression and intrinsic biosafety. This design eliminates the need for separate containment and expression modules, reducing genetic redundancy while simplifying future strain engineering. From a translational perspective, integrating therapeutic function and biosafety within a single chromosomal modification represents an attractive strategy for next-generation live biotherapeutic platforms, where genetic stability, manufacturing consistency, and regulatory compliance are increasingly regarded as complementary rather than independent design objectives.

Long-term passaging experiments suggested a stability advantage for the chromosomal chassis, but the nature of this advantage should be defined precisely. In this study, two related but distinct stability readouts were evaluated. The first was reporter-plasmid retention, assessed by monitoring maintenance of pMAX-GFP during serial non-selective propagation. Under these conditions, the chromosomal strain retained the reporter plasmid more efficiently than either the control strain carrying pMAX-GFP alone or the episomal strain carrying both pMAX-GFP and pNZ44-*hlyA*. This finding indicates improved maintenance of plasmid-borne cargo within the chromosomal chassis background, but it should not be interpreted as a direct measure of whole-strain genomic stability.

The second readout was structural maintenance of the engineered chromosomal locus after approximately 100 bacterial generations in the absence of antibiotic selection. PCR amplification of the edited region, together with Sanger sequencing of junction-spanning and internal amplicons, supported the continued presence of the Δ*ilvC*::*hlyA* insertion at the expected genomic site after prolonged passaging. These data provide structure-level evidence that the engineered locus remained associated with the expected chromosomal region throughout the experimental period.

Importantly, Sanger sequencing of PCR amplicons spanning the engineered locus provided additional structure-level support for maintenance of the integrated cassette after serial passaging. Sequencing reads generated from integration-specific and internal *hlyA* primers confirmed the presence of the 5′ donor-side junction, internal regions of the *hlyA* coding sequence, and the downstream donor-side region, indicating preservation of the overall architecture of the integrated *ilvC::hlyA* locus after approximately 100 bacterial generations. Although these data do not constitute exhaustive nucleotide-level validation of the entire insert, they strengthen the conclusion that the chromosomal modification remained stably associated with the expected genomic region under non-selective conditions. In the context of live biotherapeutic development, this is particularly relevant because preservation of locus structure is as important as retention of phenotype, especially for strains intended to undergo repeated propagation during manufacturing and preclinical testing. While these findings provide initial evidence of chromosomal stability over approximately 100 bacterial generations, future studies extending to 100–1000 generations using whole-genome sequencing together with single-cell analyses will be important to assess genome-wide stability, population heterogeneity, and long-term maintenance of the engineered chassis under conditions relevant to manufacturing and therapeutic application.

An important objective of this work was to determine whether reducing *hlyA* copy number through chromosomal integration would compromise production of biologically active LLO. Despite the expected decrease in gene dosage relative to multicopy plasmid expression, the chromosomal strain exhibited β-haemolytic activity comparable to that of the episomal construct. These findings demonstrate that chromosomal expression produced sufficient LLO to preserve functional membrane-disrupting activity, suggesting that high-copy-number expression may not be necessary to support the pore-forming function required for bacterial escape from the endosomal compartment during bactofection. From an engineering perspective, this observation is particularly relevant because excessive expression of pore-forming proteins could potentially increase bacterial fitness costs or host-cell damage without necessarily improving intracellular delivery.

**Figure 7:**
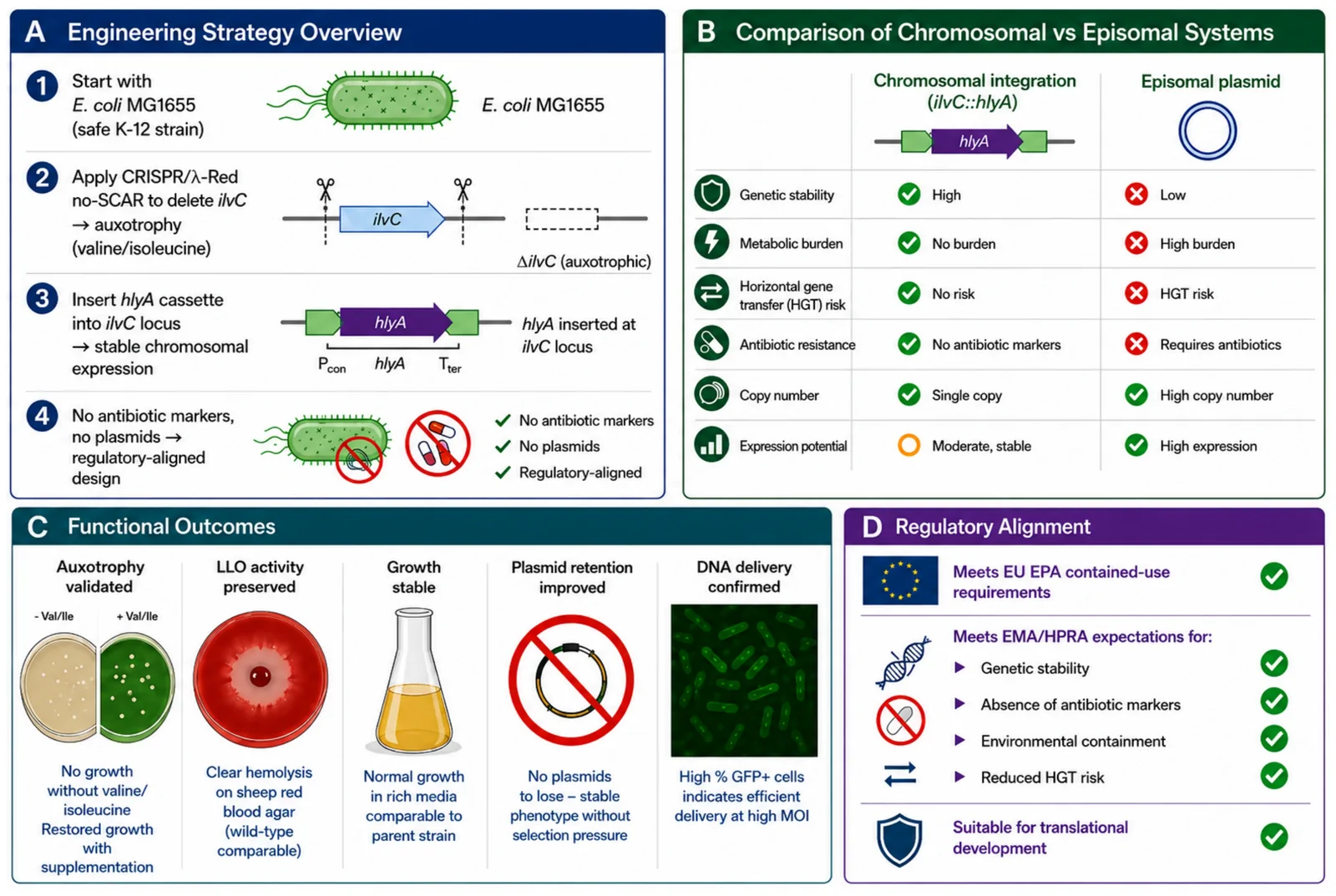
Conceptual overview of the engineering strategy and functional validation of a chromosomally integrated, auxotrophic *E. coli* MG1655 chassis for stable and biosafe DNA delivery.

The combination of stable chromosomal expression and preserved LLO activity represents an important advantage for bacterial DNA-delivery platforms. Whereas plasmid-based systems frequently rely on high gene copy number to maximise protein production, chromosomal integration offers the opportunity to achieve a more balanced expression profile while avoiding many of the limitations associated with multicopy plasmids. Such genetic streamlining is expected to improve manufacturing consistency by reducing population heterogeneity and minimising the progressive loss of engineered traits during strain propagation and scale-up. These characteristics are particularly desirable for live biotherapeutic products, in which batch-to-batch reproducibility, genetic stability, and manufacturing robustness constitute key components of quality-by-design principles and regulatory expectations. Taken together, these findings support a streamlined engineering strategy in which chromosomal integration simultaneously improves genetic stability, preserves therapeutic functionality, and incorporates intrinsic biosafety. This integrated design concept is summarised in Figure 7 and provides the framework for interpreting the functional bactofection results discussed below.

The bactofection experiments provide preliminary evidence that chromosomal expression of *hlyA* can support intracellular DNA delivery while preserving host-cell viability under conditions of increased bacterial challenge. At a multiplicity of infection (MOI) of 300, both the episomal and chromosomal systems generated comparable proportions of GFP-positive RAW 264.7 macrophages, indicating that chromosomal integration did not compromise intracellular delivery under moderate infection conditions. These observations demonstrate that genomic expression of *hlyA* was sufficient to maintain the essential membrane-disrupting activity required for bacterial escape from the endosomal compartment and subsequent release of the reporter plasmid into the host-cell cytoplasm.

This interpretation is supported by the preserved haemolytic activity observed for the chromosomal strain despite the expected reduction in *hlyA* gene copy number. Rather than maximising toxin production, effective bactofection likely depends on achieving an appropriate level of membrane permeabilisation that enables intracellular release of the therapeutic cargo without compromising host-cell survival. Similar principles have been proposed for other bacterial delivery systems, in which excessive expression of virulence-associated proteins can reduce delivery efficiency by inducing rapid host-cell death before adequate intracellular processing of the therapeutic payload occurs [13]. Although the precise relationship between LLO dosage and intracellular delivery efficiency remains to be determined, the present findings suggest that chromosomal expression may represent an effective strategy for optimising this balance.

From a translational perspective, maintaining host-cell viability is particularly important for nucleic acid delivery platforms. Successful bactofection requires not only bacterial internalisation and endosomal escape but also sufficient survival of the recipient cell to permit transcription and expression of the delivered genetic material. Consequently, preservation of viable target cells represents an integral component of delivery efficiency rather than merely a reduction in cytotoxicity. The qualitative improvements observed for the chromosomal strain under higher bacterial exposure therefore suggest that chromosomal engineering may contribute to a broader functional operating window for bacterial DNA-delivery systems, particularly under conditions in which increased bacterial uptake is required to achieve therapeutically relevant intracellular delivery.

Beyond its biological implications, the chromosomal architecture also offers practical advantages for future therapeutic development. Elimination of an additional therapeutic plasmid simplifies the genetic composition of the bacterial chassis, reduces dependence on antibiotic selection during strain maintenance, and minimises opportunities for horizontal transfer of recombinant DNA. Combined with auxotrophy-mediated biocontainment and sustained genetic stability, these characteristics establish a chassis that is inherently better aligned with current expectations for the development of live biotherapeutic products. Rather than relying solely on high-level expression of therapeutic genes, the present strategy illustrates how rational genomic engineering can simultaneously improve functional performance, biosafety, and manufacturing robustness.

The rapid clinical development of live biotherapeutic products (LBPs), exemplified by the recent regulatory approval of Rebyota™ and Vowst™, highlights the increasing importance of bacterial chassis that combine therapeutic functionality with genetic stability, intrinsic biosafety, and predictable manufacturing performance. Regulatory agencies, including the U.S. Food and Drug Administration (FDA) and the European Medicines Agency (EMA), increasingly emphasise comprehensive evaluation of genomic stability, environmental persistence, horizontal gene transfer, and biological containment during the development of engineered microbial therapeutics. In this context, the chromosomal Δ*ilvC::hlyA* platform described here was designed to incorporate several of these desirable characteristics directly into its genetic architecture by combining stable chromosomal expression, auxotrophy-mediated biocontainment, and elimination of therapeutic plasmid dependence within a single engineered chassis. Although regulatory qualification requires substantially broader preclinical and clinical evaluation, this design is conceptually aligned with current expectations for next-generation live biotherapeutic products.

Future studies should therefore focus on validating the platform under biologically and environmentally relevant conditions. These investigations should include quantitative assessment of auxotrophic containment and reversion frequency, evaluation of environmental persistence under nutrient-limited conditions, whole-genome sequencing following extended propagation, and optimisation of chromosomal expression for therapeutic nucleic acid delivery. In parallel, studies employing clinically relevant DNA, RNA, or genome-editing payloads in appropriate animal models will be essential to determine whether the advantages observed in vitro translate into improved therapeutic efficacy and safety in vivo.

Overall, the present work provides proof-of-concept evidence that a single-step chromosomal engineering strategy can be used to combine auxotrophy-mediated biocontainment with functional *hlyA* insertion in *E. coli* MG1655. The resulting chromosomal chassis preserved haemolytic activity, maintained a functional auxotrophic phenotype, and displayed favourable stability-associated characteristics relative to the episomal comparator, while remaining compatible with exploratory intracellular DNA delivery. These findings support further development of an integrated chromosomal LBP chassis that reduces dependence on plasmid-borne therapeutic expression modules and incorporates biosafety by design. Future work should include quantitative assessment of LLO expression and cytotoxicity, biological replication of bactofection experiments, broader genomic stability analyses, and in vivo evaluation of containment and delivery performance before translational claims can be made.

## 5. Conclusions

This study demonstrates the feasibility of combining therapeutic-gene integration and auxotrophy engineering within a single chromosomal modification to generate a genetically stable and biosafe bacterial chassis for live biotherapeutic applications. By replacing plasmid-based *hlyA* expression with targeted chromosomal integration at the *ilvC* locus, the engineered strain incorporates intrinsic biocontainment, eliminates reliance on antibiotic-dependent maintenance of the therapeutic module, and provides a streamlined genetic architecture for bacterial DNA delivery.

Comparative analyses showed that chromosomal integration preserved functional listeriolysin O activity, maintained bacterial growth under supplemented laboratory conditions, improved long-term genetic stability, and supported intracellular DNA delivery. Although the bactofection experiments were performed as proof-of-concept studies without independent biological replication and therefore require further validation, the observed findings suggest that chromosomal expression of *hlyA* may provide a favourable balance between delivery performance and host-cell preservation.

More broadly, this work establishes a rational chassis-engineering strategy in which therapeutic functionality, intrinsic biocontainment, and genetic stability are integrated within a single genomic modification. Rather than addressing biosafety and regulatory considerations as independent downstream objectives, this approach incorporates these design features during strain construction, providing a simplified framework for the development of genetically modified live biotherapeutic products. Future validation in replicated in vitro and in vivo models, together with comprehensive assessment of long-term genomic stability and therapeutic efficacy, will be essential to define the translational potential of this platform.

## Figures and Tables

**Table 1:** Primers used for PCR and Sanger validation of chromosomal *hlyA* integration.

Primer/Read Name	Primer Sequence (5’-3’)	Primary Use
hly_integration_fwd	GAA TGT GAC CGC GTG TAA GC	5′ donor/junction validation
hlyAF	AAA CTT CGG AAT CAG TG	Internal *hlyA* sequencing
hly_fwd_3	AAT TCC GCC AGG GTC AGA TG	Internal *hlyA* sequencing
hly_fwd_2	CTT CAC TGA TTG CGC CGA AG	Internal/distal *hlyA* sequencing
hly_integration_rev	TTC CAC TAT CGC AGC CAT GG	3′ donor/junction validation
hly_rev_2	AGG AAG GTT TTC AGA TCG CGT	Internal *hlyA* sequencing

**Table 2:** Summary of Sanger sequencing support across the integrated *ilvC*::*hlyA* cassette.

Region of the Donor Construct	Interpretation
5′ homology arm	Confirmed by the upstream integration read.
5′ junction	Confirmed by the 5′ junction-spanning read.
*hlyA* proximal/internal region	Confirmed by internal *hlyA* reads.
*hlyA* distal region	Confirmed by distal and internal *hlyA* reads.
*hlyA*–3′ donor-side junction	Confirmed by the 3′ junction-spanning read.
Downstream donor	Confirmed by downstream donor reads.

**Table 3:** Comparative bactofection performance of episomal and chromosomal *hlyA* expression systems in RAW 264.7 macrophages. Values represent descriptive flow-cytometry measurements obtained following bactofection at MOI 300 and 500.

Parameter	Control	Episomal (MOI 300)	Chromosomal (MOI 300)	Episomal (MOI 500)	Chromosomal (MOI 500)
Viability (%)	74.4	32.4	34.9	4.9	23.0
GFP-Positive cells (%)	3.4	45.5	42.6	10.7	37.5
Mean FSC-A	132,272	75,580	82,213	55,596	68,533
Mean GFP Fluorescence (488-2A)	310	554	577	551	584

Abbreviations: MOI, multiplicity of infection; FSC-A, forward scatter area. Control, untreated RAW 264.7 macrophages; Episomal, RAW 264.7 macrophages bactofected with *E. coli* MG1655 carrying *pNZ44-hlyA* and pMAX-GFP; Chromosomal, RAW 264.7 macrophages bactofected with *E. coli* MG1655 Δ*ilvC*::*hlyA* carrying pMAX-GFP.

## Data Availability

The data supporting the findings of this study are not publicly available due to ongoing analyses and project dependencies but can be obtained from the corresponding author upon reasonable request.

## Supplementary Materials

The following supporting information can be downloaded at: https://www.mdpi.com/article/10.3390/pharmaceutics18080915/s1, File S1: Supplementary Methods (S1. no-SCAR genome editing strategy; S2. Donor construct design; S3. PCR verification of chromosomal integration; S4. Plasmid curing; S5. Auxotrophy assay) and Supplementary DNA Sequence. Figure S1: pMax-GFP transformation validation. Figure S2: Comparative β-haemolytic activity of chromosomal and episomal *hlyA* expression systems. Figure S3: Growth kinetics of *E. coli* MG1655 strains carrying chromosomal or plasmid-based *hlyA* constructs. Figure S4: PCR verification of chromosomal integration of *hlyA* into the *ilvC* locus.

## Abbreviations

The following abbreviations are used in this manuscript:

LBP	Live Biotherapeutic Product
GMM	Genetically Modified Microorganism
ATMP	Advanced Therapy Medicinal Product
GTMP	Gene Therapy Medicinal Product
EPA	Environmental Protection Agency
HPRA	Health Products Regulatory Authority
EU	European Union
BCAA	Branched Chain Amino Acid
LLO	Listeriolysin O
MOI	Multiplicity of Infection
CFU	Colony Forming Unit
PCR	Polymerase Chain Reaction
OD_600_	Optical Density at 600 nm
AUC	Area Under the Curve
DMEM	Dulbecco’s Modified Eagle Medium
FBS	Foetal bovine serum
GFP	Green Fluorescent Protein
CRISPR	Clustered Regularly Interspaced Short Palindromic Repeats
