# Supplementary material for: Single-Step Chromosomal Engineering Integrates Biocontainment and Therapeutic Function for Regulatory-Oriented Live Biotherapeutic Chassis Design
Source: Pharmaceutics. 2026 Jul 24;18(8):915. doi: 10.3390/pharmaceutics18080915 (PMC13517021; doi:10.3390/pharmaceutics18080915)
Supplement: Supplementary file 1 [file pharmaceutics-18-00915-s001.zip › pharmaceutics-4280075-supplementary.pdf]

SUPPLEMENTARY MATERIAL

Supplementary Methods

S1. No-SCAR genome editing strategy

Chromosomal integration of the *hlyA* expression cassette into the *ilvC* locus of *Escherichia coli* MG1655 was performed using the no-SCAR genome-editing system, which combines λ-Red recombineering with CRISPR/Cas9-mediated counterselection to generate seamless chromosomal modifications without leaving antibiotic resistance markers or scar sequences.

Briefly, chemically competent MG1655 cells carrying the pCas9 and pKDsgRNA plasmids were transformed with the synthetic donor DNA containing the *hlyA* expression cassette flanked by homologous regions corresponding to the chromosomal sequences immediately upstream and downstream of the *ilvC* coding sequence. Recombinant colonies generated following homologous recombination were enriched through CRISPR/Cas9 counterselection and subsequently screened by PCR. Candidate integrants were further confirmed by Sanger sequencing before plasmid curing.

S2. Donor construct design

The donor construct was designed to replace the complete chromosomal *ilvC* coding sequence with the *Listeria monocytogenes hlyA* expression cassette through homologous recombination.

The synthetic donor DNA was commercially synthesized (GenScript, Piscataway, NJ, USA). Primer sequences used for PCR validation and Sanger sequencing are provided below together with the complete donor DNA sequence.

Sequence features:

- **5′ homology arm:** 1,000 bp
- ***hlyA* gene:** 1,590 bp
- **3′ homology arm:** 1,000 bp
- **Primer *hlyA* integration FWD:** GAATGTGACCGCGTGTAAGC

- **Primer *hlyA*_ Rev:** TTCCACTATCGCAGCCATGG
- **Primer *hlyA* integration Rev:** AAACTTCGGCGCAATCAGTG

GAATGTGACCGCGTGTAAGCCGCTAACCGGTTGGCTGCGGATGTTTAAATCAGAGAG
AGAAGACCGAAATCGGAGTTCATCGTGAATCCATTAGACTTAGAAAATATCGGGTCGT
AACGTTGTGTTATTACTTTTTCAGAGTTTACTAAGGAGAGGTTTACTCGTGGCGATTAA
TTTCAGCCCTAAGGTTGGTGAAATACTGGAATGCAACTTTGGTAACTATCCTGTTAGC
CAAAATGGACCGTTCAGCACGACGTATTACGATGGCCGCATTCCTCCTGAAATGATAA
AAAACAGGTTAGTTGTCGTGCTCAACGGAAAAATTAATGGCAACGCATGCATCGTCG
TTCCATTATCAACCACTCGCGACCAGACAAGTTAAAACGTGGAATGCATGTTGAAATA
GCCAGTAATGTTATCAATGACCTGCAATTCTTTGATCAGCAGATTCGTTGGGCCAAAG
CCGACCTGGTACAACAAGTCAGTCGAAACAGATTAAACAGAGCAAGGACTTACAGA
GGGTATCTTAATCAATGTTTGCCACATGAGTTGGTTGCAGACATACAGAGGGCGGTCA
TCAAGTCAATCAATGCCATATCGCTCATAAATTGACAATAAAATAACCATCCCCGATAA
TGACTTTGTAGCCCATAGAGGCCCTAAGTTTTCTCCCCTTGAGGGACCTGCGAAGCCC
GGATTTTCCGGGCTTTTGCCATATATGTACCAGCCACTCCCTGACTGACAATTACGTTA
GCATCTGCGGATCATGCTAAAATCGCCGCCTGACAATTATTCACTCAAGGCACGATCA
TGGCAAAAACAGCAGCAGCACTGCATATCCTTGTAAAAGAAGAGAAACTGGCTCTG
GATCTTCTCGAGCAGATTAAGAACGGGGCCGATTTCGGCAAGCTGGCGAAGAAACAC
TCCATTTGCCCATCAGGCAAACGCGGCGGTGATTTAGGTGAATTCCGCCAGGGTCAG
ATGGTTCCGGCGTTCGATAAAGTGGTTTTCTCTTGTCCGGTACTGGAGCCGACCGGCC
CGCTGCACACCCAGTTCGGATATCACATCATTAAGGTGCTGTACCGCAACTAATAGCA
AGGCCTTCTCCAGGAGAAGGCCTTGAGTGTTTTCTCCCTCTCCCTGTGGGAGAGGGT

CGGGGTGAGGGCATCAGCGCACTTAACCCGCAACAGCAATACGTTTCATATCTGTC

ATATAGTTATTCGATTGGATTATCTACTTTATTACTATATTTCGGATAAAGCGTGGTGCCC

CAGATGGAGATATTTCTATTTTTCACAAGTGGTAAGTTCCGGTCATCAATTACCGTTCT

CCACCATTCCCAAGCTAAACCAGTGCATTCTTTAGCGTAAACATTAATATTTCTCGCGT

TACCTGGCAAATAGATGGACGATGTGAAATGAGCTAGCTTGCTTTTATTGTTTTCGCTC

CAGTTTTTATGTTGAACAATTTCGTTACCTTCAGGATCATAATTTACTTCATCCCAAGA

AATGTTGAATTGAGCAACGTATCCTCCAGAGTGATCGATGTTAATTTTTCCATCTGTAT

AAGCTTTTGAAGTTGTTTCAATATATTCTGAGTTGTTTTTAATAACAGCTAATTCATTGT

CTTTTAGGAAGTTTGTTGTATAAGCAATGGGAACTCCTGGTGTTTCTCGATTAAAAGT

AGCGCCTTTTTTCAAAATATCGCGTAAGTCTCCGAGGTTGCCGTCGATGATTTGAACT

TCATCTTTTGCGGAACCTCCGTAAATTACGGCTTTGAAGGAAGAATTTTTGATGATATT

TGTTAGTTCTACATCACCTGAGACAGATTTTCCGCTTACGGCAGCATCAAAAGCAGCT

TTTACTTTAGTACTATGGGAATTAGTTGATAATTTCAAATAAACTTGACGGCCATACGC

CACACTTGAGATATATGCAGGAGGATTTTCTGCATTCACTCCAAGCGCTTGCAACTGC

TCTTTAGTAACAGCTTTGCCGAAAAATCTGGAAGGTCTTGTAGGTTCATTAACATTCA

CGTTATAGTAAATTTGTTTAAAACTAATGACTTCTTCTTGCATTTTCCCTTCACTGATTG

CGCCGAAGTTTACATTCAAGCTATTATTTACAGCTTTAAATGCTGTACCAAATTTCGCA

ATTAATTGTGATTCACTGTAAGCCATTTCGTCATCATAATCAATTTTTGCACTTACATTT

GGATAAGCTTGAGCATATTTTTCATTCCATCTTTCCACTAATGTATTTACTGCGTTGTTA

ACGTTTGATTTAGTGGCATTTTTTACAACGATTTTATTGTCTTGATTAGTCATACCTGGC

AAATCAATGCTGAGTGTTAATGAATCACGTTTTACAGGGAGAACATCTGGTTGATTTT

CTACTAATTCCGAATTCGCTTTTACGAGAGCACCTGGATAGGTTAGGCTCGAAATTGC

ATTCACAACTTGAATGTCTGCATTATTTTGATTGATGGATTTCTTCTTTTTCTCCACAAC

AATATATTCATTTCCATCTTTGTAACCTTTTCTTGGCGGCACATTTGTCACTGCATCTCC

GTGGTATACTAATACATTGTTTTTATTGTAATCCAATCCTTGTATATACTTATCGATTTCA

TCCGCGTGTTTCTTTTCGATTGGCGTCTTAGGACTTGCAGGCGGAGATGCTGGTGGTG

CCATGGATGAAATTGAATTTTCTTTATTGAATGCAGATGCATCCTTTGCTTCAGTTTGTT

GCGCAATTGGTAGACTAACTAATATAAGTGTAATAAAAACTAGCATTATTTTTTTCATG

GTGATTCCTCGTGATGTTGTGCTTCTTATTGTTCGGTTCGCGTTTGCGAGATTGAATTC

ACTATATGACAGGAAATTTATTGCGGAAATTGATATATTCACAACGTCACATTGCAATT

TTTGCAACGTCAACATCGAGGGCTGTCCCTGTGGATTTACGCGATCTGAAAACCTTCC

TGCATCTGGCGGAAAGCCGCCATTTTGGCCGCAGCGCGGGCGATGCACGTTAGCC

CATCCACGCTCTCACGGCAGATTCAGCGCCTGGAAGAAGATCTCGGTCAGCCGCTGT

TTGTGCGCGATAACCGCACGGTGACGCTGACTGAAGCGGGCGAAGAGCTGCGCGTTT

TCGCCCAGCAAACGCTGTTGCAGTATCAGCAGTTGCGCCACACCATCGATCAGCAAG

GGCCGTCGCTCTCTGGCGAATTACATATCTTCTGCTCGGTGACCGCTGCCTACAGCCA

TCTGCCGCCGATTCTGGATCGCTTCCGCGCGGAACACCCGTCGGTGGAGATTAAACTT

ACTACTGGTGATGCGGCAGATGCGATGGAAAAGGTGGTCACTGGTGAAGCGGATCTG

GCGATTGCGGGTAAACCGGAAACCTTGCCCGGCGCAGTGGCGTTTTCGATGCTGGAG

AATCTGGCAGTAGTGCTGATTGCCCCCGCGCTGCCCTGCCCGGTGCGTAATCAGGTGT

CGGTAGAGAAGCCGGACTGGTCAACGGTGCCGTTTATTATGGCCGATCAGGGGCCGG

TACGCCGCCGCATTGAACTGTGGTTTCGACGCAATAAAATCAGTAACCCGATGATTTA

CGCCACGGTTGGCGGGCATGAAGCGATGGTATCGATGGTGGCACTCGGCTGTGGCGT

GGCATTGTTGCCGGAAGTGGTGCTGGAAAACAGCCCCGAACCGGTGCGTAACCGCG

TGATGATTTTAGAGCGCAGCGATGAGAAAACGCCGTTTGAGCTGGGCGTGTGTGCGC

AGAAAAAGCGGCTACATGAGCCGCTAATTGAGGCATTCTGGAAGATTTTGCCGAACC

ACAAATGACGTTGTCGCGCGGGTAGGCCTGATAAGCGAAGCGCTATCAGGCATTTTTC

CCTAACCCGCCAAAAAGAACCTGAACGCCGGGTTATTGGTTTCGTCGTGGCAATCGT

AGCCCAGCTCATTCAGCCGGGTTTCGAAATCCGGTTCATGGTCGCCAAGTTCGAACG

CCGCCAGTACGCGCCCGTAGTCGGTGCCATGGCTGCGATAGTGGAA

S3. PCR verification of chromosomal integration.

Correct chromosomal integration was confirmed using two complementary PCR strategies. The first PCR employed primers located outside the recombination region to amplify the complete chromosomal integration locus, producing the expected amplicon of approximately 4.1 kb. The second strategy employed combinations of external chromosomal primers and internal *hlyA*-specific primers to verify both chromosomal junctions independently. Expected PCR products of 2,107 bp and 2,736 bp confirmed correct insertion of the *hlyA* cassette into the *ilvC* locus. Representative agarose gel electrophoresis demonstrating the expected PCR products together with the relative primer positions is presented in Figure S4**.**

S4. Plasmid curing.

Following successful chromosomal integration, genome-editing plasmids were removed by serial passaging under non-selective conditions. Recombinant colonies were cultured repeatedly in antibiotic-free LB medium at 37 °C to eliminate the temperature-sensitive editing plasmids. Loss of the editing plasmids was confirmed before subsequent phenotypic and molecular characterization of the engineered strain.

S5. Auxotrophy assay.

Functional disruption of *ilvC* was evaluated using growth assays in M9 minimal medium supplemented either with or without branched-chain amino acids. Wild-type MG1655 and chromosomal Δ*ilvC::hlyA* strains were cultured in M9 medium in the absence or presence of isoleucine and valine (50 mg/L each). Growth of the engineered strain occurred only under supplemented conditions, confirming successful disruption of the branched-chain amino acid biosynthetic pathway and establishment of the intended auxotrophic phenotype.

Supplementary DNA Sequence

Supplementary Figures

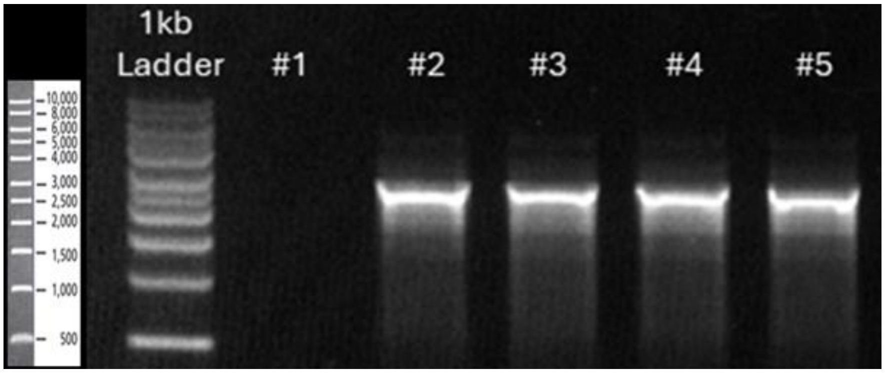

Figure S1. pMax-GFP transformation validation. Agarose gel electrophoresis of plasmid DNA from *E. coli* MG1655 constructs carrying GFP and *hlyA* variants. Lane 1: *E. coli* ATCC negative control. Lane 2: GFP miniprep positive control. Lane 3: MG1655 [pMAX-GFP]. Lane 4: MG1655 [pNZ44-*hlyA*; pMAX-GFP]. Lane 5: MG1655 Δ*ilvC::hlyA* [pMAX-GFP].

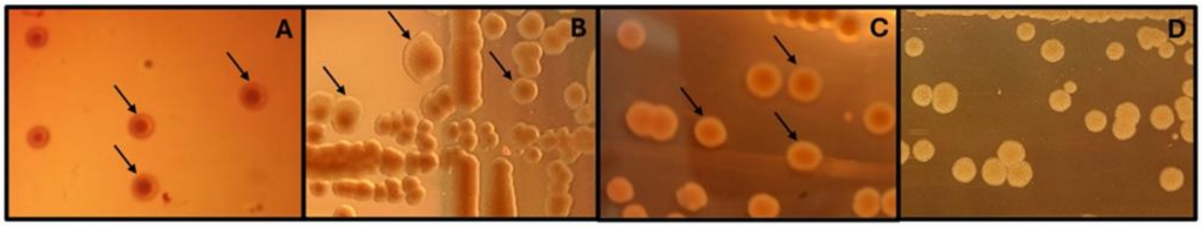

Figure S2. Comparative β-haemolytic activity of chromosomal and episomal *hlyA* expression systems. Representative Columbia agar plates supplemented with 5% defibrinated sheep blood following 24 h incubation at 37 °C. Clear β-haemolytic zones were observed around colonies of *Listeria monocytogenes* (positive control), MG1655 [pNZ44-*hlyA*], and MG1655 Δ*ilvC::hlyA*, whereas no haemolysis was detected for parental MG1655. Comparable qualitative β-haemolytic phenotypes were observed for both chromosomal and episomal expression systems. Images are representative of three independent experiments.

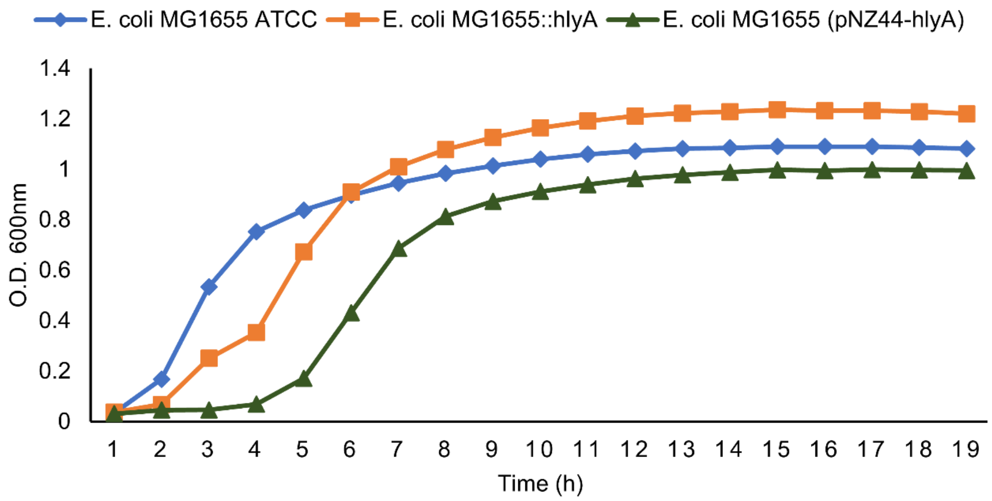

Figure S3. Growth kinetics of *Escherichia coli* MG1655 strains carrying chromosomal or plasmid-based *hlyA* constructs. Representative growth curves comparing parental MG1655, chromosomal Δ*ilvC::hlyA*, and episomal MG1655 [pNZ44-*hlyA*] strains cultured in LB broth at 37 °C. Optical density (OD600) was monitored hourly for 19 h.

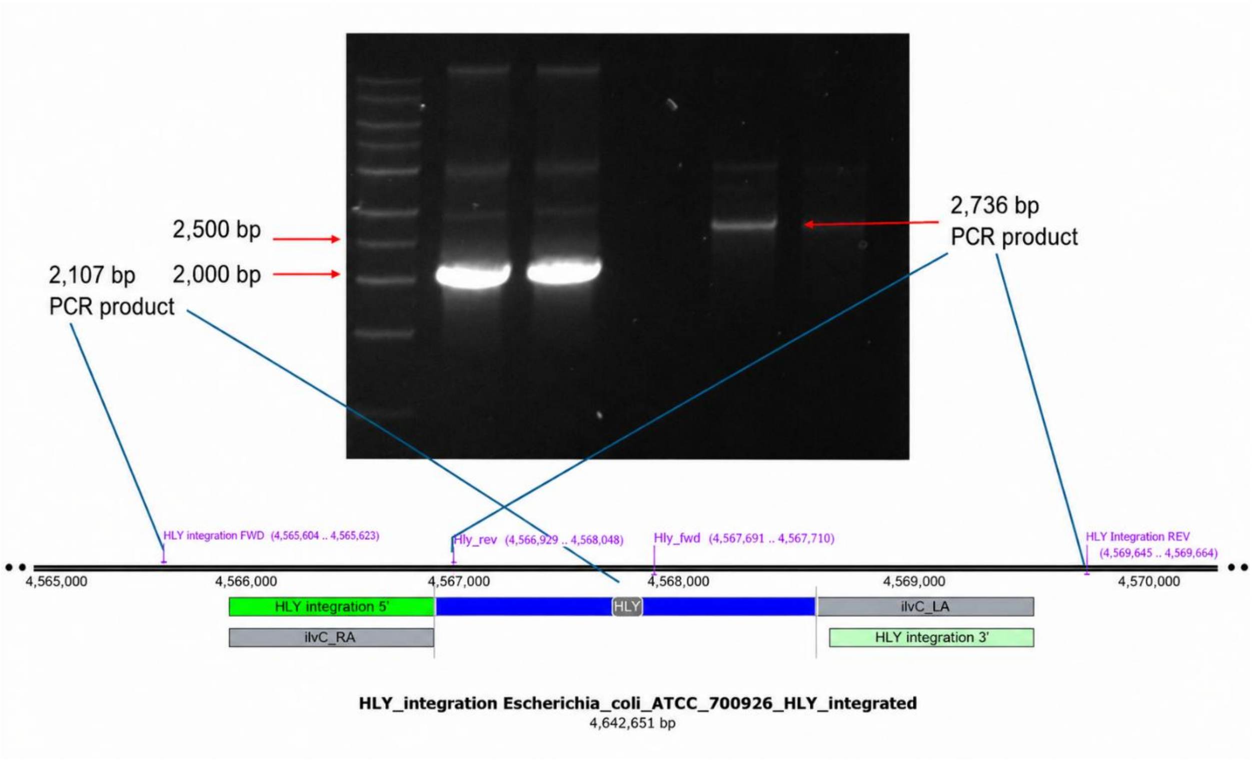

Figure S4. PCR verification of chromosomal integration of *hlyA* into the *ilvC* locus. Representative agarose gel electrophoresis showing PCR products obtained using combinations of

external chromosomal primers and internal *hlyA*-specific primers to verify both chromosomal junctions. Expected PCR products of 2,107 bp and 2,736 bp confirmed correct integration of the *hlyA* cassette into the *ilvC* locus. The lower panel illustrates the genomic organization of the engineered locus together with primer positions and expected amplification products.
